# Functionally Relevant Residues of Cdr1p: A Multidrug ABC Transporter of Human Pathogenic *Candida albicans*

**DOI:** 10.4061/2011/531412

**Published:** 2011-04-27

**Authors:** Rajendra Prasad, Monika Sharma, Manpreet Kaur Rawal

**Affiliations:** Membrane Biology Laboratory, School of Life Sciences, Jawaharlal Nehru University, New Delhi 110067, India

## Abstract

Reduced intracellular accumulation of drugs (due to rapid efflux) mediated by the efflux pump proteins belonging to ABC (ATP Binding Cassette) and MFS (Major Facilitators) superfamily is one of the most common strategies adopted by multidrug resistance (MDR) pathogenic yeasts. To combat MDR, it is essential to understand the structure and function of these transporters so that inhibitors/modulators to these can be developed. The sequence alignments of the ABC transporters reveal selective divergence within much conserved domains of Nucleotide-Binding Domains (NBDs) which is unique to all fungal transporters. Recently, the role of conserved but divergent residues of *Candida* Drug Resistance 1 (*CDR1*), an ABC drug transporter of human pathogenic *Candida albicans*, has been examined with regard to ATP binding and hydrolysis. In this paper, we focus on some of the recent advances on the relevance of divergent and conserved amino acids of CaCdr1p and also discuss as to how drug interacts with Trans Membrane Domains (TMDs) residues for its extrusion from MDR cells.

## 1. Introduction

The pathogenic *Candida albicans *accounts for approximately 50–60% causes of candidiasis particularly in immuno-compromised human patients. But the infections caused by non-*albicans* species, such as *C. glabrata*, *C. parapsilosis*, *C. tropicalis*, and *C. krusei* are also common particularly in neutropenic patients and neonates [1–4]. Of note, recently, the incidences of *albicans* and non-*albicans* species of *Candida* acquiring resistance to antifungals (particularly to azoles) have increased considerably which poses problems towards its successful chemotherapy [5–7]. On one hand, to combat antifungal resistance, search for better drugs with newer targets is underway; on the other hand, *Candida* cells have evolved a variety of strategies to develop resistance to common antifungals.

**Figure 1:**
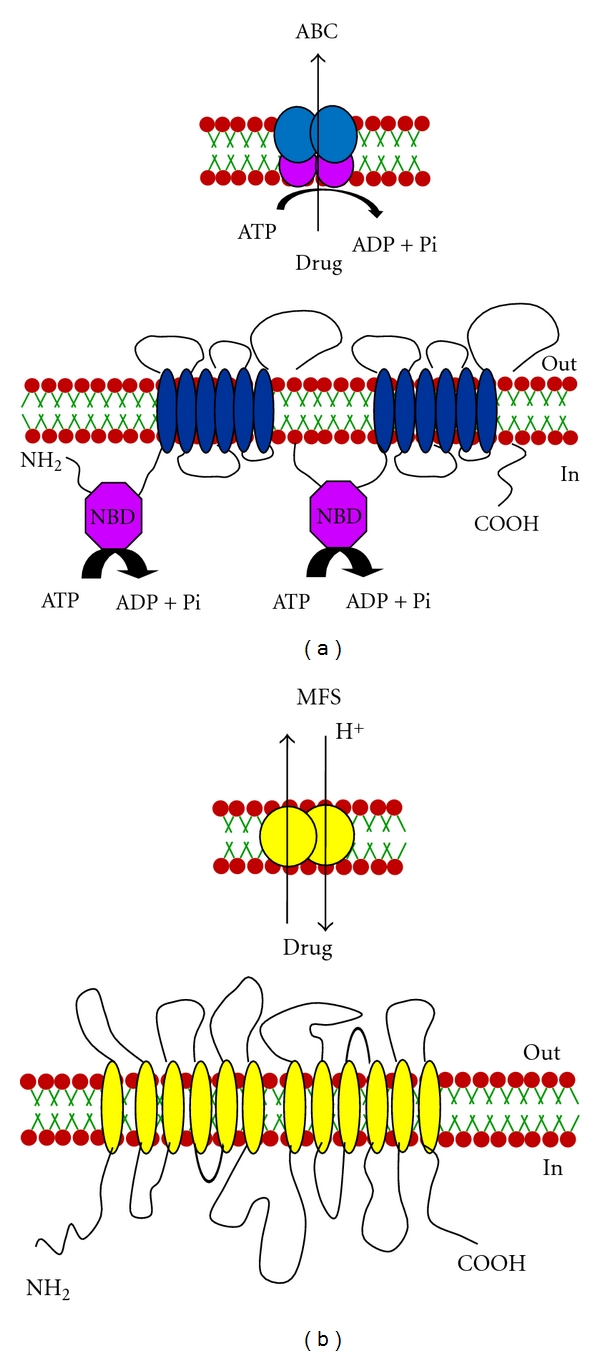
A cartoon representation of (a) ABC and (b) MFS transporters of Candida. The topology of ABC and the MFS transporters depicted here have the (NBD-TMS_6_)_2_ and the (TMS)_12_ (Transmembrane Segments) arrangements, respectively. The NBDs (Nucleotide-Binding Domains) of the ABC transporters are responsible for the hydrolysis of ATP, which facilitates drug extrusion while the MFS transporters utilize proton gradient to expel drugs.

The main mechanisms of antifungal resistance to azoles include alterations in ergosterol biosynthetic pathway by an overexpression of *ERG11* gene which encodes the drug target enzyme 14*∝*-demethylase or by an alteration in target enzymes (point mutations) [3, 8, 9]. Reduced intracellular accumulation of drugs (due to rapid efflux) is another prominent mechanism of resistance in *Candida* cells [10]. Most commonly, genes encoding drug efflux pumps belonging to ABC (ATP binding cassette) and MFS (Major Facilitator) superfamilies of proteins are overexpressed in azole resistant *Candida* isolates which abrogates intracellular accumulation leading to enhanced tolerance to drugs (Figures 1(a) and 1(b)).

## 2. Efflux Pumps

Since ABC and MFS transporters are among the major players that contribute to azole resistance in clinical isolates of *Candida*, there is a spurt in research on all aspects of these genes and their encoded proteins [6, 7]. In this context, considerable attention is being paid to the structural and functional aspects of these proteins, which in turn could lead to better strategies for designing modulators/inhibitors of these pumps. The genome of *C. albicans* possesses 28 ABC and 95 MFS proteins; however, only ABC transporters CaCdr1p and CaCdr2p and MFS transporter CaMdr1p are known to be multidrug transporters which play major role in drug extrusion from resistant strains. In this review, we begin with a discussion on the structure and function of ABC proteins and then focus on the role of some of the critical amino acid residues of CaCdr1p in drug transport. For brevity, we have excluded MFS drug transporters from our discussion.

## 3. Structure and Function of ABC Efflux Proteins

**Figure 2:**
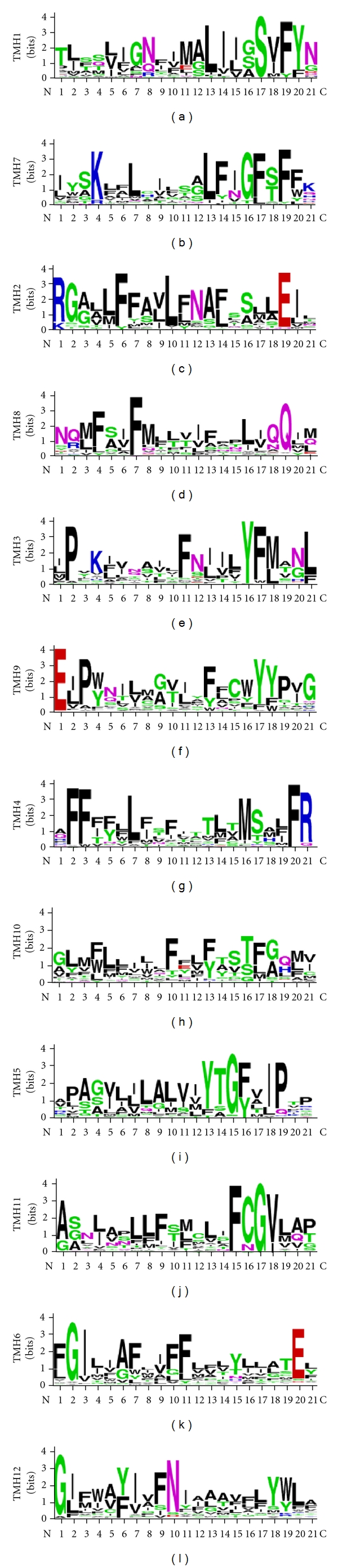
Sequence logos of CaCdr1p transmembrane segment (TMSs) residues with other fungal PDR transporters. Each logo consists of stacks of symbols, one stack for each position in the sequence. The scale indicates the certainty of finding a particular amino acid at a given position and is determined by multiplying the frequency of that amino acid by the total information at that position. The residues at each position are arranged in order of predominance from top to bottom, with the highest frequency residue at the top. The height of symbols within the stack indicates the relative frequency of each amino acid at that position. Colors such as green defines polar, blue correspond to basic, red to acidic, black to hydrophobic, and violet represent the amino acids that have polar amide group.

**Figure 3:**
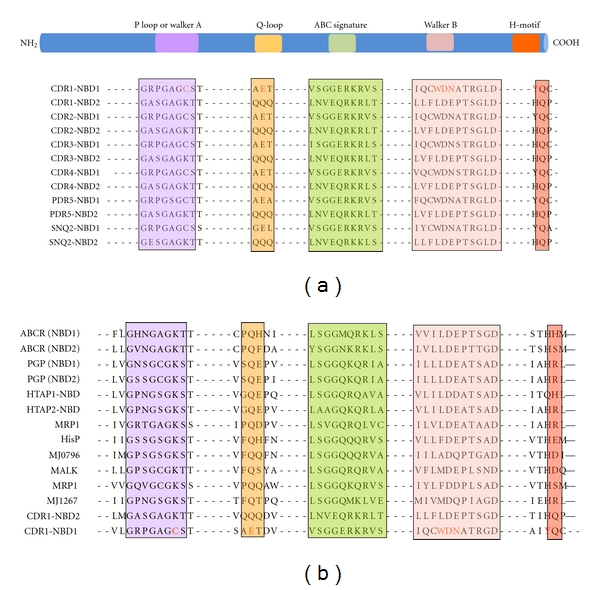
Sequence alignment of the conserved motifs from fungal ABC transporters. Comparison of the sequence alignment of the walker A, Q-loop, signature C, Walker B, and H-loop motifs of N- and C-terminal NBDs (NBD1 and NBD2) of CaCdr1p with known (a) fungal and (b) nonfungal ABC transporters. Conserved but unique residues are highlighted.

ABC proteins are generally made up of two transmembrane domains (TMDs), each consisting of six transmembrane segments (TMS) and two cytoplasmically located nucleotide-binding domains (NBDs) which precedes each TMD (Figures 1(a) and 1(b)), [11, 12]. While it appears that several TMSs associate together to form the substrate binding site(s), this alone is probably not sufficient for substrate transport across the membrane bilayer. Vectorial transport of these substrates requires energy from the hydrolysis of ATP carried out at the NBDs. Given their varied roles and the greatly differing characteristics of substrates that members of this superfamily of proteins seem to efflux, it is hardly surprising that despite the overall conservation of the domain architecture of TMDs, their primary sequences are significantly different (Figure 2). On the other hand, NBDs of ABC transporters which power drug transport are highly conserved both in terms of primary structure and architecture (Figure 3). 

## 4. Candida Drug Resistance 1 (CDR1)

*CDR1* of *C. albicans*, the first ABC efflux pump characterized in any known pathogenic yeast, was isolated as a gene implicated in conferring resistance to cycloheximide in a *PDR5* disruptant hypersensitive strain of *S. cerevisiae* [13]. *CaCDR1* codes for a protein of 1501 amino acid residues (169.9 kDa), with a topology similar to that of ABC proteins Pdr5p and Snq2p of *S. cerevisiae.* On the other hand, its topology mirrors that of *STE6*, a -mating pheromone transporter of *S. cerevisiae*, as well as of the human MDR1 and CFTR. Despite a high structural and functional similarity between CaCdr1p and ScPdr5p, some distinct functional features tend to distinguish them. For example, both genes share overlapping specificities for cycloheximide and chloramphenicol but *CaCDR1* affects sensitivity to oligomycin while neither amplification nor disruption of Sc*PDR5* alters susceptibilities to this mitochondrial inhibitor [13]. It is worth mentioning that some of the close homologues of *CaCDR1* in *C. albicans* are also functionally distinct. For example, CaCdr2p that exhibits 84% identity with CaCdr1p has a distinct drug resistance profile [14]. The overexpression or deletion of *CaCDR3* and *CaCDR4*, the homologues of *CaCDR1* and *CaCDR2* interestingly do not affect drug susceptibilities of yeast cells [15]. The hydropathy plots of CaCdr1p and CaCdr3p show that both the proteins have similar topological arrangements where the hydrophilic domain containing the NBDs precedes the hydrophobic TMS [16]. The only apparent difference between the two proteins appears to be in the C-terminal where CaCdr3p has an extended loop connecting TM11 and TM12. In addition, there is stretch of 21 amino acids in the C-terminal of CaCdr3p which are absent in CaCdr1p [16]. Keeping in view, the importance of these regions in drug binding and transport, the subtle differences in the primary structures of these proteins could be responsible in governing their substrate specificity, hence only enabling some of them (CaCdr1p and CaCdr2p) to bind and transport drugs [17, 18].

**Figure 4:**
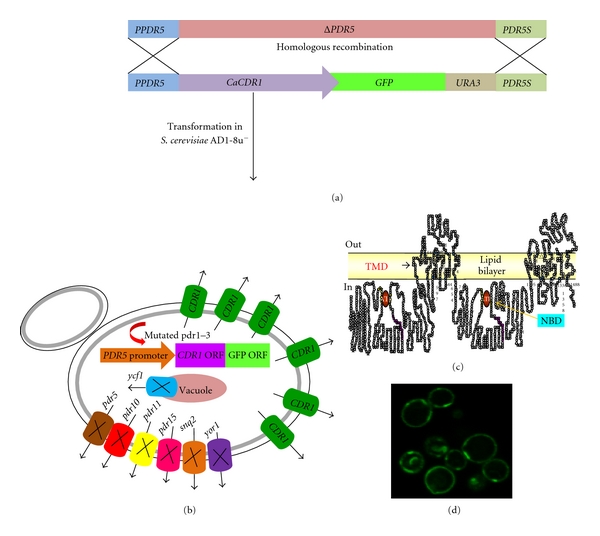
Overexpression of CaCdr1p in a heterologous system. (a) Strategy showing the cloning and transformation of *CaCDR1-GFP* in *S. cerevisiae. *(b) Pictorial representation of the host AD1-8u^−^ showing the Deleted ABC pump proteins (*pdr5*, *pdr10*, *pdr11*, *pdr15*, *snq2*, *yor1*, *ycf1*) and the hyper expressed *CaCDR1-GFP*. (c) Topology of CaCdr1p. (d) Localization of CaCdr1-GFP in the host strain AD1-8u^−^. The rimmed green fluorescent depicts overexpressing GFP tagged CaCdr1p.

To study the MDR proteins, a heterologous hyperexpression system is used where GFP tagged CaCdr1p/CaMdr1p has been stably overexpressed from a genomic *PDR5* locus in a *S. cerevisiae *mutant AD1-8u^−^ [19]. The host AD1-8u^−^ developed by Goffeau's group [20] was derived from a *Pdr1-3* mutant strain with a gain of function mutation in the transcription factor Pdr1p, resulting in constitutive hyperinduction of the *PDR5* promoter [19]. In previous studies, we have confirmed that GFP tagging of CaCdr1p (*CaCDR1-GFP*) and CaMdr1p (*CaMDR1-GFP*) did not impair its expression and the functional activity of the proteins [21, 22].  Figure 4 summarizes the strategy used for the expression of CaCDR1-GFP under *ScPDR5* promoter.

## 5. How Does CaCDR1 Power Drug Efflux?

The characteristic feature of CaCdr1p or of any other ABC drug transporter is that they utilize the energy of ATP hydrolysis to transport variety of substrates across the plasma membrane. The conserved NBDs located at the cytoplasmic periphery are the hub of such an activity. The NBDs of all ABC transporters, irrespective of their origin and nature of transport substrate, share extensive amino acid sequence identity within typical motifs [12]. For example, NBDs of ABC transporters have a *β*-sheet subdomain containing the typical Walker A and Walker B motifs, as an essential feature of all ATP requiring enzymes [23], along with an *α*-helical sub-domain that possesses the conserved ABC signature sequence. NBD domain sequences possess certain conserved amino acid stretches, which are considered to be critical for its functionality [24]. These include: the Walker A, with a consensus sequence GxxGxG*K*S/T, where “*x*” represents any amino acid, the Walker B motif, that is, hhhhD, where “*h*” represents any aliphatic residue, and an ABC signature, LSGGQQ/R/KQR. Structural and biochemical analyses of NBDs show that the well-conserved lysine residue of Walker A motif binds to the *β*- and *γ*-phosphates of ribonucleotides and plays a critical role in ATP hydrolysis [24]. Mutations of this lysine residue have been shown to reduce or abolish the hydrolysis activity and in some cases impair nucleotide binding [24]. Interestingly, though N-terminal NBD of CaCdr1p contains the conserved Walker A (GRPGAG*C*ST) and B (IQCWD) motifs, and an ABC signature sequence (VSGGERKRVSIA) [25], the commonly occurring lysine residue within the Walker A motif is replaced with a cysteine. This replacement appears to be a unique feature of N-terminal NBDs (N-NBD) of most of the known fungal ABC-type transporters. In addition, degeneracy in Q loop and Walker B also exist in N-NBDs of all fungal transporters including in CaCdr1p. Notably, the C-terminal NBDs display degeneracy only in signature motifs (discussed later).

To ascertain the role of the uncommon cysteine of Walker A, active N-NBD of CaCdr1p was cloned and overexpressed and the soluble domain protein was purified and characterized. It was observed that an evolutionarily divergent Cys193 of Walker A of N-NBD was critical for ATP hydrolysis. The relative contribution of both the N- and C-terminal NBDs in ATP binding, hydrolysis, and transporter activity of native CaCdr1p (full protein) was examined wherein the atypical Cys193 of Walker A of N-NBD (C193K) and conserved Lys901 (K901C) in the Walker A of C-terminal NBD (C-NBD) were replaced [26]. The drug resistance profile of CaCdr1p mutant variant cells harboring C193K or K901C gave interesting insights into the functioning of the two NBDs. The cells expressing K901C showed enhanced hypersensitivity to drugs as compared to C193K variant which displayed partial sensitivity to select drugs. These observations clearly established that the two NBDs respond asymmetrically to the substitution of conserved residues of their respective Walker A motifs. The functional asymmetry of NBDs in CaCdr1p was also illustrated in another study where swapping of NBDs resulted in non-functional CaCdr1p chimeras and thus suggested that the two NBDs are not identical and nonexchangeable [27]. Interestingly, in the case of human P-gp, a close homologue of CaCdr1p, the issue of functional symmetry of two NBDs remains contentious. Approaches addressing this issue in P-gp provide data both in favor [28, 29] and against [30–32] functional asymmetry. This is in contrast with many other ABC transporters, for which there is evidence that the two NBDs, although highly similar in sequence, may adopt different functional roles in the transport cycle [33].

**Figure 5:**
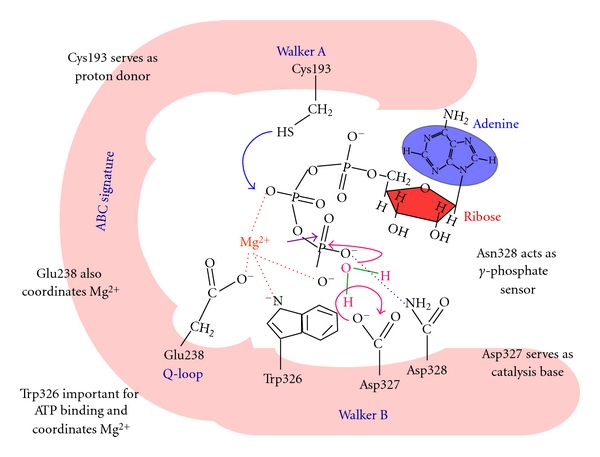
A hypothetical model depicting the N-terminal active site of CaCdr1p. The role of various residues involved in the catalytic mechanism for ATP hydrolysis by the N-NBD of CaCdr1p the details are discussed in the text.

Any functional asymmetry observed in the intact transporter is probably not entirely due to inherent properties of the NBD, and presumably also reflects either differences in the rate of hydrolysis or the effects of interdomain interactions. Two other residues of the N-NBD from CaCdr1p are also found to be important for domain functioning. As depicted in Figure 5, the unusual Trp326 in the Walker B motif of N-NBD, which is unique and conserved in all fungal transporters, is important for ATP binding and for the accompanying conformational change [34]. Thus, although the mutant with W326A appears capable of ATP hydrolysis, it does so with a much higher *K*_*M*_ value, indicating that the docking of the substrate in the binding pocket has been altered by the mutation. However, the protein appears capable of near-normal function in cells expressing the full-length protein carrying W326A mutation, implying that the conformational change that normally occurs upon ATP docking cannot by itself be responsible for the cross-talk by the domain with the TMDs. While the highly conserved Asp327 of N-NBD is shown to be the catalytic carboxylate in the context of other ABC transporters, in N-NBD of CaCdr1p, it does not appear to mediate catalysis *via* interaction with Mg^2+^ as is normally expected for similar transporters [34]. It has been shown that due to spatial proximity, fluorescence resonance energy transfer (FRET) takes place between Trp326 of Walker B and MIANS [2-(4-maleimidoanilino) naphthalene-6-sulfonic acid] on Cys193 of Walker A motif. These critical amino acids are positioned within the nucleotide-binding pocket of N-NBD to bind and hydrolyze ATP. The results show that both Mg^2+^ coordination and nucleotide binding contribute to the formation of the active site. The entry of Mg^2+^ into the active site causes the first large conformational change that brings Trp326 and Cys193 in close proximity to each other. It was also demonstrated that besides Trp326, typical Glu238 in the Q-loop also participates in coordination of Mg^2+^ by N-NBD. A second conformational change is induced when ATP, but not ADP, docks into the pocket. The unique Asn328 does sensing of the *γ*-phosphate of the substrate in the extended Walker B motif, which is essential for the second conformational change that must necessarily precede ATP hydrolysis.

It has been possible to deduce a picture of the catalytic mechanism for ATP hydrolysis by the N-NBD of CaCdr1p (Figure 5). The metal ion approaches the nucleotide binding pocket and forms a *π*-stacking interaction with the delocalized electron cloud of Trp326 in Walker B. This induces a large conformational change in the protein, bringing Cys193, Glu238, Trp326, Asp327, and Asn328 closer into the nucleotide binding pocket. At this point, the metal ion is sufficiently far from the MIANS on Cys193 to have no effect on its fluorescence intensity. However, Trp326 and MIANS on Cys193 are within 16 Å of each other at this point. ATP approaches with its phosphates directed towards the pocket. As in other ATPases, it may be assumed that the *β* and *γ*-phosphates also coordinately bind the metal ion and their negative charges are considerably masked. This is important since in the absence of the metal ion, the nucleotide does not dock into the active site. While other residues may also be involved in stabilizing the nucleotide within the pocket, Asn328 certainly acts as a sensor for the *γ*-phosphate. This induces the second conformational change within the protein. Asp327 which acts as a catalytic base abstracts a proton from a water molecule, that is, part of the Mg-ATP complex present in the active site. The hydroxyl ion, thus, formed in turn attacks at the *β*-phosphate allowing it to, in turn, abstract a proton from the –SH of Cys193. The consequence of this is to simultaneously weaken the phosphodiester bond between *β*- and *γ*-phosphates, allowing the latter to leave. Once ATP is hydrolyzed, Asn328 no longer senses the *γ*-phosphate, and the conformation relaxes back to a more open one allowing ADP to leave (Figure 5). 

The data thus far unequivocally show that the N-NBD of CaCdr1p and by extension those of other fungal transporters have evolved so as to use their unique substitutions to perform the task of ATP binding and hydrolysis. While it is not yet clear what evolutionary advantage these typical sequence variations might provide to the organisms, it is becoming more and more evident that it has mechanistic implications for the protein. We are yet to understand how the N-NBD works in conjunction with the C-NBD to give rise to a functional drug transporter. Does working in tandem require the ABC Signature sequence of one NBD to participate in ATP binding by the other, as is seen in other ABC transporters? Like in the N-NBDs, the ABC signature sequences of CaCdr1p and other fungal transporters too appear to have diverged away from that of other ABC transporters. Whether this is so as to compensate for the substitutions in their N-NBDs or whether they have evolved a new mechanism for coming together for ATP hydrolysis and drug efflux is a question worth examining.

**Figure 6:**
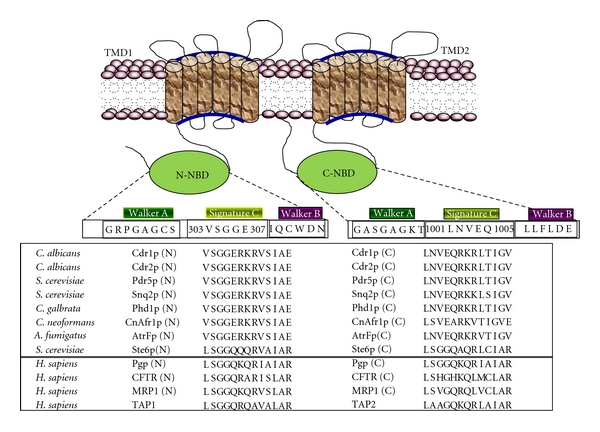
Topology of CaCdr1p and sequence alignment of signature motifs from various ABC transporters. The sequence alignment of signature motif residues in NBDs with those from other nucleotide-binding domains of some known ABC transporters is shown.

Signature motifs are other domains which are the hallmark sequences of NBDs of ABC transporters that display highly conserved sequences across the evolutionary scale; however, there are also instances of appearance of selective divergence within this motif. For example, human ABC transporters such as TAP [35] and CFTR [36] have degenerated Signature motifs (Figure 6). In contrast, all the family members of ABC transporters of fungi, particularly of PDR subfamily display divergence in their Signature motifs. Thus, the Signature motif of N-NBD of CaCdr1p is well conserved but has C-NBD with a degenerated Signature motif (Figure 6). Our recent analysis revealed that the conserved and degenerated Signature sequences of the CaCdr1p are functionally indispensable and cannot be exchanged. This emphasizes the uncompromised asymmetry that exists between the NBDs of CaCdr1p and in other yeast ABC transporters. Similar to other ABC transporters, the well-conserved serine (S304) and glycine (G306) residues present in conserved Signature motif of N-NBD are also critical for the functioning of CaCdr1p. For example, even the substitution at the equivalent position residues of degenerated Signature motif of C-NBD with the conserved ones and vice versa does not support the function of the transporter [25]. The well-conserved glycine present at fourth position of Signature motif (LSG*G*Q) is involved in the ATP catalysis [25, 30, 36–39]. Biochemical analysis revealed that a small change at this position (G→A) results in steric hindrance between methyl group of alanine and *γ*-phosphate of ATP. If this glycine is exchanged with bulky, charged aspartate or glutamate, it leads to a complete loss of ATPase and protein activity [25, 40, 41]. The critical nature of serine and glycine in WT-CaCdr1p can also be compared with similar residue of those proteins whose crystal structures are known. The existing structural information suggests that the Signature motifs of ABC proteins; Rad50 of *Pyrococcus furiosus*, MJ1096 of *Methanocaldococcus jannaschii*, GlcV of *Sulfolobus solfataricus,* Sav1866 of *Staphylococcus aureus,* mouse CFTR, HlyB, and MalK of *E.coli*, are involved in the head to tail ATPase site formation with the Walker A and Walker B motifs of the opposite NBDs, sandwiched with ATP molecules wherein the Signature motif is a “sensor” for an ATP *γ*-phosphate in the opposing domain [24, 42–49]. Based on the conserved nature of these motifs, it is reasonable to speculate that in CaCdr1p, the conserved S304 and G306 of NBD1 probably fall within close proximity of the ATP binding site. In addition, divergent residues present in C-NBD Signature region are also equally important and may be part of the ATPase site as well. However, it still requires experimental validation.

Additionally, it is shown that in addition to highly conserved and critical S304 and G306 residues, the equipositional residues N1002 and E1004 of degenerated Signature motif of C-NBD of WT-CaCdr1p have also evolved to be functionally essential. Notably, pairs of residue like V303, G305 of N-NBD and L1001, V1003, Q1005 of C-NBD Signature motif though part of otherwise conserved Signature sequences has apparently no functional relevance. These residues when replaced with either alanines or with its equipositional substitutes continued to show phenotypes similar to cells expressing WT-CaCdr1p.

Functional nonequivalence in the NBDs of ABC proteins of yeast is the result of variations in the conserved motifs (Walker A, Walker B, H-loop and Signature motif). These variations in N-NBD may have evolved in response to degenerated Signature motif of C-NBD. Thus, in CaCdr1p, both canonical and noncanonical ATP binding sites are formed similar to TAP and CFTR proteins. Recently, Ernst et al. hypothesized that in Pdr5p of *S. cerevisiae*, one ATP molecule catalyzed at the canonical active site may be sufficient to reset the TMDs whereas the second non-canonical site (regulatory site) may be engaged to serve as platform for keeping domains in dimeric form (inward facing) [49, 50].

## 6. CaCDR1 Extrudes Structurally Unrelated Substrates

The range of CaCdr1p substrates varies enormously and includes structurally unrelated compounds such as azoles, lipids, and steroids (Table 1). This promiscuity towards substrates is a characteristic feature of most ABC-type drug transporters and, hence, makes their functionality all the more complex to understand. Expectedly, predicting the residues involved in substrate binding without high-resolution structural data is a challenge. Yet, using a combination of biochemical assays along with site-directed mutagenesis, it has been possible to partially dissect the substrate binding pockets of CaCdr1p wherein role of some of the TMS amino acids in drug extrusion is becoming apparent [17, 18, 21].

## 7. Nature of Substrate Binding

Experiments with purified CaCdr1p have conclusively shown that ATP binding to CaCdr1p is not a prerequisite for drug binding and both the mechanisms of drug and ATP binding result in specific conformational changes which take place independent of each other [51]. A direct link between the ability of CaCdr1p to translocate fluorescent glycerophospholipids and efflux drugs has also been demonstrated [51]. Considering chemically diverse substrates which are expelled by CaCdr1p, the exact number of residues involved in drug binding and transport is far from understood.

As mentioned earlier, the CaCdr1p was overexpressed as a GFP-tagged fusion protein in a heterologous hyperexpression system and was characterized for drugs and nucleotide binding [52]. Iodoarylazido prazosin (IAAP, a photoaffinity analogue of P-gp substrate, prazosine) and azidopine (a dihydropyridine photoaffinity analogue of P-gp modulator, verapamil) were shown specifically to bind with *CaCDR1-GFP*. Interestingly, IAAP binding with CaCdr1p-GFP was competed out by molar excess of nystatin while azidopine binding could only be competed out by miconazole, thus, highlighting the possibility of different drug binding sites for the two analogues [52]. Gauthier and coworkers [52] have also shown that membranes prepared form CaCdr1p and CaCdr2p expressing cells are capable of binding the photoaffinity analogue of rhodamine 123 (^125^I) iodoaryl azido-rhodamine 123 (IAARh123) and that both N-terminal and C-terminal halves of CaCdr2p contribute to rhodamine binding [52].

**Figure 7:**
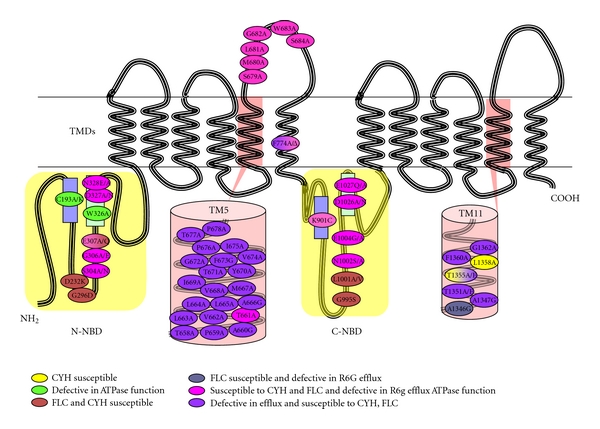
Cartoon of CaCDR1 protein depicting the location and phenotype of the mutated amino acids. Residues important for the resistance to CYH have been marked in yellow, defective in ATPase function in green, susceptible to FLC and CYH in blue, susceptible to FLC and defective in R6G efflux in grey, susceptible to CYH and FLC and defective ATPase function in pink, defective in efflux and susceptible to CYH, FLC in purple.

**Figure 8:**
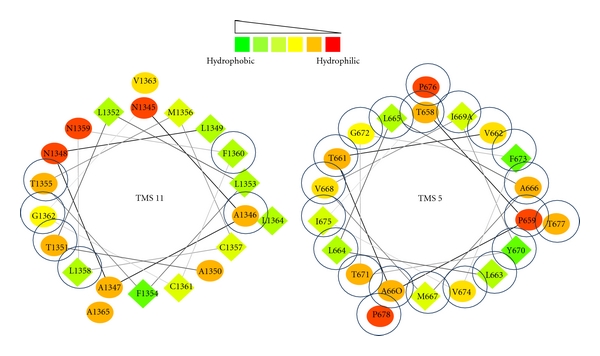
Helical wheel projection of TMS 5 and TMS11 of CaCdr1p. Helical wheel projection of the protein sequence was constructed by the EMBOSS PEPWHEEL program. This displays the sequence in a helical representation as if looking down the axis of the helix. The hydrophilic residues as circles, hydrophobic residues as diamonds. Hydrophobicity is color coded as well: the most hydrophobic residue is green, and the amount of green is decreasing proportionally to the hydrophobicity, with zero hydrophobicity coded as yellow. Hydrophilic residues are coded red with pure red being the most hydrophilic residue, and the amount of red decreasing proportionally to the hydrophilicity. The mutations that affected drug resistance are circled blue.

To understand the mechanism of drug transport mediated by CaCdr1p, a battery of its mutant variants that drastically affect various stages of drug extrusion have been generated (Figure 7). Amino acids of two of the twelve TMSs of CaCdr1p were subjected to alanine scanning wherein all the residues were replaced with alanines. The alanine scanning of TMS 11 of CaCdr1p showed that at least seven residues which were critical for determining substrate specificity and drug transport were clustered on the hydrophilic face of the *α* helical projection of TMS11 [18]. In contrast, alanine scanning of TMS 5 highlighted the importance of all 21 residues in drug transport and substrate specificity [17]. Based on the drug susceptibility pattern, the mutant variants of the TMS 5 could be grouped into two categories. The mutants belonging to first category exhibited sensitivity to all the tested drugs while the mutants placed in the other category showed intermediate level of resistance. While the ATPase activity and drug binding were largely unaffected, rhodamine 6G (R6G) and [^3^H] fluconazole (FLC) efflux was abrogated in all the mutant variants. Based on the competition experiments with the molar excess of substrates during R6G efflux, we could identify residues which may be specific for interactions with miconazole (MCZ), itraconazole (ITR), and ketoconazole (KTC) and those which were common to all the three azoles. Notably, FLC which is also a substrate of CaCdr1p did not compete with R6G efflux; hence implying that CaCdr1p has independent binding sites for this azole. All the mutant variants display uncoupling between ATPase activity and drug transport, and thus TMS 5 of CaCdr1p not only appears to impart substrate specificity but probably also acts as a communication helix. What constitutes the substrate/drug binding pocket and how TMS 5 interacts with other helices of CaCdr1p are some of the issues that remain to be resolved (Figure 8).

Together, studies so far suggest that the drug binding sites in CaCdr1p are scattered throughout the protein and probably more than one residue of different helices are involved in binding and extrusion of drugs. However, there is still insufficient information available to predict where exactly the most common antifungals, such as azoles bind and how they are extruded. However, such studies should pave the way for future investigations related to the dynamics of substrate selection and may improve our approach in the design of new inhibitors/modulators of drug transporter for clinical applications.

## 8. Concluding Remarks

The drug transporters belonging to either ABC or MFS superfamily of proteins are the main contributors of azole resistance in pathogenic *C. albicans. *In this regard, CaCdr1p, a major ABC multidrug transporter, has been widely studied. CaCdr1p like its sister fungal homologues is unique in terms of variant sequences present in otherwise conserved domains in NBDs. It is established that each unique substitution in CaCdr1p and by extension of other fungal ABC transporters have dedicated role in ATP binding and hydrolysis and, thus, are essential for drug efflux. Why fungal transporters alone have evolved and retained these divergent domain based amino acid substitution is not understood. But these unique residues also provide an opportunity to develop novel modulators or inhibitors of these efflux pump proteins.

## Figures and Tables

**Table 1:** Substrates and inhibitors of CaCDR1 substrates.

Substrates	Fluconazole, ketoconazole, voriconazole, Itraconazole, miconazole, lipids, steroids, R6G, cycloheximide, rhodamine 123, cerulenin, trifluoperazine, nigericin, tamoxifen, verapamil, cycloheximide, propanil, diuron, linuron, disulfiram, anisomycin, doxorubicin, 4-nitroquinoline –N-oxide, benomyl, yohimbine HCl, quinidine, etoposide, chlorobromuron, vinblastine, tamoxifen, gefitinib, fluphenazine, topotecan, daunorubicin, DM-11, AT-12 niguldipine, dexamethasone, berberine, terbinafine, tritylmazole	[7, 53]

Inhibitors/modulators	Milbemycins, enniatin, FK506, FK520, unnarmicins, curcumin, disulfiram	[54–57]

## Abbreviations

ABC: ATP binding cassette
MFS: Major facilitator superfamily
IAAP: Iodoarylazido prazosin
NBD: Nucleotide-binding domain
TMSs: Trans membrane segments
TMDs: Trans membrane domains
CDR: *Candida* drug resistance protein
GFP: Green fluorescence protein.
